# Appearance-Based Multimodal Human Tracking and Identification for Healthcare in the Digital Home

**DOI:** 10.3390/s140814253

**Published:** 2014-08-05

**Authors:** Mau-Tsuen Yang, Shen-Yen Huang

**Affiliations:** Department of Computer Science & Information Engineering, National Dong-Hwa University, No. 1, Sec. 2, Da-Hsueh Rd., Shoufeng, Hualien 974, Taiwan; E-Mail: 610221034@ems.ndhu.edu.tw

**Keywords:** home healthcare, human detection, human tracking, human identification

## Abstract

There is an urgent need for intelligent home surveillance systems to provide home security, monitor health conditions, and detect emergencies of family members. One of the fundamental problems to realize the power of these intelligent services is how to detect, track, and identify people at home. Compared to RFID tags that need to be worn all the time, vision-based sensors provide a natural and nonintrusive solution. Observing that body appearance and body build, as well as face, provide valuable cues for human identification, we model and record multi-view faces, full-body colors and shapes of family members in an appearance database by using two Kinects located at a home's entrance. Then the Kinects and another set of color cameras installed in other parts of the house are used to detect, track, and identify people by matching the captured color images with the registered templates in the appearance database. People are detected and tracked by multisensor fusion (Kinects and color cameras) using a Kalman filter that can handle duplicate or partial measurements. People are identified by multimodal fusion (face, body appearance, and silhouette) using a track-based majority voting. Moreover, the appearance-based human detection, tracking, and identification modules can cooperate seamlessly and benefit from each other. Experimental results show the effectiveness of the human tracking across multiple sensors and human identification considering the information of multi-view faces, full-body clothes, and silhouettes. The proposed home surveillance system can be applied to domestic applications in digital home security and intelligent healthcare.

## 1. Introduction

With the advances in medical technologies, the global population is aging, and the elderly are becoming the fastest growing population sector in most developed countries. In addition to elders, toddlers and patients are also at a higher risk of falling and require continuous and long-term monitoring. There is an urgent need for an intelligent and inexpensive home surveillance system to provide home security, monitor health conditions, and detect emergencies of family members. To realize the power of these intelligent services in digital homes, one of the fundamental problems is how to detect, track, and identify people in a home environment. Wearable RFIDs can identify humans effectively, but users are forced to wear tags all the time. Alternatively, vision-based surveillance provides a natural and non-intrusive solution to human detection, tracking, and identification.

Typical smart homes deploy a variety of visual sensors (or cameras) located in and around house to monitor human activities and detect critical events. Each type of visual sensors has its own unique strengths and limitations. For example, iris scanners are very accurate in human identification but only work on stationary people within a close range. Similarly, fingerprint scanners require people to show fingers from a very short distance. Alternatively, a depth camera can resolve the problems of casting shadows and dynamic illuminations but its sensing range is limited, for example, 0.8 ∼ 4 m for a Kinect [1]. In addition, face recognition algorithms provide passive sensing but rely on high resolution facial images. As a result, typical video surveillance systems utilize omni-directional (OD) cameras to locate people and subsequently guide pan-tilt-zoom (PTZ) cameras to capture close-up facial images. However, clear facial images are unavailable when a person wears a mask or turns back to the camera intentionally. Table 1 compares the pros and cons of various sensors in a home environment.

Recently, Rice *et al.* [2] revealed that when facial features are difficult to make out, we readily use body information to identify a person. Our brains use information from a person's body size, shape, build, and stance for recognition even before we can distinctly see a face. Compared with recognition solely based on faces, they discovered that human recognition is far more accurate when both the face and body of the person are shown. In addition to human recognition, human detection and tracking can also utilize the body appearance to handle the challenging problems of multi-view variations, posture changes, shape deformations, far away views, and partial occlusions. Therefore, we propose appearance-based human detection, tracking, and identification modules that cooperate with each other seamlessly based on multimodal fusion of multi-view faces, body colors, and silhouettes captured by multiple sensors.

Several techniques have been proposed to perform multimodal fusion at various levels using different methods. Multimodal inputs can be integrated at three levels: the signal (low) level, the feature (intermediate) level, and the decision (high) level. Fusion at a higher level offers scalability and flexibility, but loses signal (or feature) correlation among modalities. Fusion at a lower level offers less interaction, but provides more simplicity because only one learning phase on the combined vector is required. Multimodal observations can be combined for an estimation using various methods such as histogram technique, multivariate Gaussian, linear weighted sum, Kalman filter, or particle filter. Also, a decision can be made by multimodal fusion using different approaches such as majority voting, artificial neural network (ANN), support vector machine (SVM), or hidden Markov model (HMM). These multimodal fusion levels and methods are usually application specific and tailor-designed according to the natures and requirements of the target problem.

**Figure 1.**
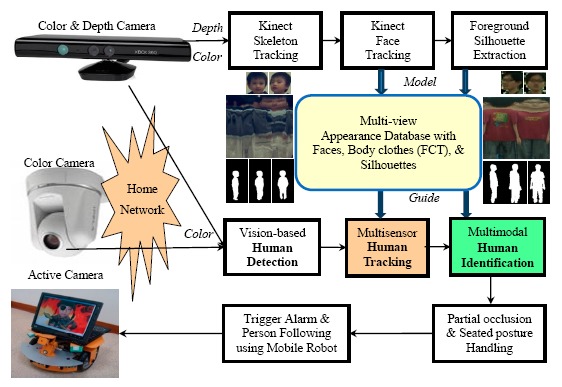
Framework of the proposed appearance-based multimodal human detection, tracking, and identification system for intelligent healthcare and security in digital homes.

The framework of the proposed human detection, tracking, and identification system is shown in Figure 1. In the modelling stage, human skeleton and face tracking are performed based on depth images captured by two Kinects installed at the home entrance. Then the captured color images are utilized to capture faces, extract body silhouettes, and construct a multi-view Flattened Cylindrical Template (FCT) in an appearance database. The FCT contains the appearance information of an individual person in an upright standing position with a view of 360°. In the guidance stage, a person is detected and tracked based on color images captured by the Kinects as well as other color cameras installed in other parts of the house. The image of the detected person is compared with each registered template in the appearance database. If a match is found, the person is identified and the corresponding template can be used to guide the subsequent human detection and tracking; otherwise the system prompts for password to further classify the person as a miss-identified family member, a guest, or an intruder. The proposed human detection, tracking, and identification system plays a key role in the application of security and healthcare in intelligent digital homes.

In this paper, we discuss the system design, development, and evaluation of the proposed appearance-based multimodal human detection, tracking, and identification system. The remaining parts of the paper are organized as follows: Section 2 reviews the relevant state-of-the-art techniques. Section 3 presents the multi-view modelling of the full-body appearance using color and depth images captured by Kinects and the construction of an appearance database. Section 4 explains the human detection and tracking using a multisensor fusion (Kinects and color cameras) based on a Kalman filter. Section 5 presents the human identification using a multimodal fusion (face, body appearance, and silhouette) based on a majority voting. Section 6 evaluates the proposed system. Section 7 handles special cases and discusses the limitations and potential applications in smart homes. Section 8 offers conclusions.

## 2. Background

In recent years, techniques for human detection, tracking and identification have progressed significantly. A RFID-based method employs either active or passive tags. Active RFID tags have a larger range but require batteries to provide power. Passive RFID tags are less expensive but only work over a short distance. Similarly, infrared (IR) or ultrasound transmitters can be installed in known positions and each person can carry an IR or ultrasound receiver that monitors signals in a range for localization. A person is located using a triangulation method based on the distance and angular measurements from at least three known locations. Generally, the IR-based methods are accurate but can suffer interference from background illumination. Relatively, the ultrasound-based approaches are cheaper but less accurate.

However, people do not feel comfortable about wearing a tag, transmitter, or receiver for a long time. To reduce disturbance, passive visual cameras provide a non-intrusive way for human detection, tracking, and identification. Sixsmith and Johnson [3] developed a smart inactivity monitor using an array-based detector, called SIMBAD, for elderly fall detections. Tao *et al.* [4] presented an infrared ceiling sensor network with binary responses to recognize eight activities including walking, tidying, watching TV, reading, taking drinks, using PC, lying, and sweeping in a home environment. Ni *et al.* [5] designed a get-up event detector to prevent potential falls in hospitals based on color and depth images captured by a Kinect. Motion and shape features from multiple modalities and channels were extracted and combined through a multiple kernel learning process. Based on a mobile robot equipped with a Kinect, Mozos *et al.* [6] used local binary patterns (LBP) and SVM to categorize indoor places including corridor, kitchen, laboratory, study room, and office. Yang and Chuang [7] adopted a Kinect to classify behaviors and assess fall risks of toddlers at home.

For human detection using a typical surveillance camera, Viola and Jones [8] proposed integral images for fast feature computations, an AdaBoost algorithm for automatic selections, and a cascade structure for efficient human detections. Dalal and Triggs [9] used histogram of oriented gradients (HOG) as features and a linear support vector machine (SVM) as a classifier for pedestrian detection and demonstrated promising accuracy. Zhu *et al.* [10] combined integral image, cascade structure, and HOG for fast human detection. Dollar *et al.* [11] proposed a fast human detector, called ChnFtrs, by extracting and integrating Harr-like features over multiple channels. They also developed a fast multi-scale variant of ChnFtrs, called FPDW [12], by using a sparsely sampled image pyramid to approximate features at intermediate scales. Benenson *et al.* [13] modified FPDW to avoid resizing the input monocular images at multiple scales and provided human detection at 50 frames per second (FPS). By exploiting geometric information using stixel estimations from stereo images, they achieved 135 FPS in a CPU + GPU enabled computer.

For vision-based human tracking, Mean-Shift [14] is a non-parametric method to find the mode of a probability distribution function (PDF). It can be applied to visual tracking by creating a PDF in the new frame based on a target model, and performing an iterative algorithm to find the peak of the PDF near the object's last position. However, the Mean-Shift algorithm only worked well on targets with static PDFs. CamShift [15] extended the Mean-Shift to handle dynamic PDFs by updating the target model based on the color histogram of the object in the previous frame, and solve scaling problem by adjusting the search window size based on the updated PDF. To model the appearance of both object and its background dynamically, Collins *et al.* [16] developed an online feature selection mechanism using a two-class variance ratio to discriminate between a tracked object and its surrounding background. Babenko *et al.* [17] presented an online multiple instance learning algorithm, called MILTrack, by extracting positive and negative examples as an adaptive appearance model for object tracking. Kalal *et al.* [18] proposed a tracking-learning-detection (TLD) framework with a pair of computerized experts that can learn from missed detections and false alarms.

Humans can be identified visually by face recognition if the detected person faces the camera within a close range. A geometric approach creates a facial signature by measuring distances between key features to capture a unique facial profile for each face. Alternatively, a photometric approach analyzes the variance of faces over a high-dimensional vector space to form a basis set of facial images. To perform dimension reduction, Eigenfaces [19] employed principal component analysis (PCA) while Fisherfaces [20] adopted linear discriminant analysis (LDA). In addition to human identification, a relevant problem, called human re-identification, is to identify a specific person across disjoint camera views and to recognize if a person has been observed over a network of cameras. It is a challenging problem due to changes in points of view, background, illumination, pose deformation, and visual occlusion. Gandhi and Trivedi [21] proposed a panoramic appearance map (PAM) as a compact signature to match people observed in different camera views. Prosser *et al.* [22] formulated the re-identification as a ranking problem and developed an Ensemble RankSVM.

Generally, tracking-based algorithms can generate a smooth trajectory of an object by estimating its motion, but they require initialization and can accumulate drift error during run-time. On the other hand, detection-based algorithms can estimate the object location in every frame independently. However, a detector requires an offline training stage and cannot detect unknown objects. Unlike these methods, we proposed appearance-based human detection, tracking, and identification modules which cooperated seamlessly and benefitted from each other by using a multimodal fusion of facial images, body colors, and silhouettes across multiple sensors in home environments.

## 3. Human Modelling of Face, Appearance and Silhouette Based on Kinects

**Figure 2.**
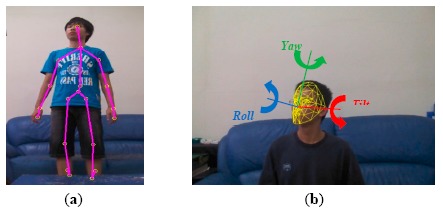
Human tracking using a Kinect. (**a**) Skeleton tracking; (**b**) Face tracking.

The proposed system modelled multi-view human appearances (faces, body colors, and silhouettes) semi-automatically using a set of the latest consumer market depth cameras, called Kinects. A Kinect captured both color and depth images at thirty frames per second with 640 × 480 resolution. The distance between a three-dimensional point and the camera is called depth (denoted as *z*). A pixel in a depth image indicated the calibrated depth of the pixel's corresponding three-dimensional point in the scene. For the human skeleton tracking, each depth image was segmented into a dense probabilistic body part labeling so that a human body was divided into thirty-one parts [23]. The body parts were defined to be spatially localized near twenty skeletal joints, hence the three-dimensional locations of the skeletal joints can be determined by back-projecting these inferred parts into a world space. As shown in Figure 2a, a complete skeleton was represented by a sixty-dimensional vector containing three-dimensional coordinates of twenty skeletal joints. In the modelling stage, the face, body appearance, and silhouette of each family member should be modelled and registered in the appearance database. As shown in Figure 7, two Kinects were installed at opposite sides in a living room to facilitate the modelling process.

### 3.1. Modelling of Face

In addition to the skeleton tracking, the Kinect's face tracking algorithm can determine the location and three-dimensional pose of a face in real-time. With the availability of two head joints (the head and the neck) in the tracked skeleton, the rough location of the face in the captured images was determined. The Kinect's face tracker extended color-based Active Appearance Model (AAM) to incorporate depth information [24]. Based on both color and depth images, the Kinect's face tracker detected 87 contour points along facial parts (as shown in Figure 2b) as well as additional 13 non-contour points including eye centers, mouth corners, a nose center, and a bounding box around the head. By registering the tracked facial features with a three-dimensional facial model, the head pose was estimated and represented by three rotational angles: tilt, yaw, and roll (as shown in Figure 2b). In the modelling stage, family members were asked to stare at the Kinect at a short distance (1.0 ∼ 2.0 m) so clear facial images can be captured and recorded in the appearance database. As shown in Figure 1, at least two face templates (front and 45° view) were stored in the appearance database for each registered person. Assuming that a human face is symmetric horizontally, the face template in −45° view can be simply obtained by mirroring that in +45° view.

### 3.2. Modelling of Body Appearance

**Figure 3.**
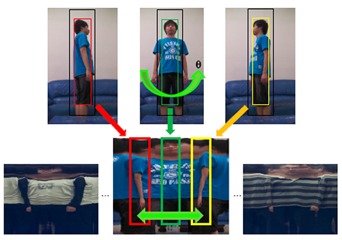
FCT-based human identification and tracking.

Whenever a person passed the living room, multi-view full-body color images were captured and the skeletal information was analyzed simultaneously by two Kinects. Then the multi-view full-body appearance information was compacted and stored in a template image, called the Flattened Cylindrical Template (FCT), in the appearance database. As shown in Figure 3, the FCT combined the captured images of a person from various viewpoints and covered the appearance of a full body in 360°. The FCT was constructed by an image mosaicking process that aligned and stitched one vertical slice in the captured color images at a time along the tracked skeletal spine. The aforementioned head pose angles can also provide alignment cues in FCT construction. As shown in Figure 1, at least one constructed FCT was stored in the appearance database for each registered person. Whenever the registered person was identified by face recognition later on but wearing a different set of clothes, a new FCT was constructed and added to the appearance database automatically.

### 3.3. Modelling of Body Silhouette

**Figure 4.**
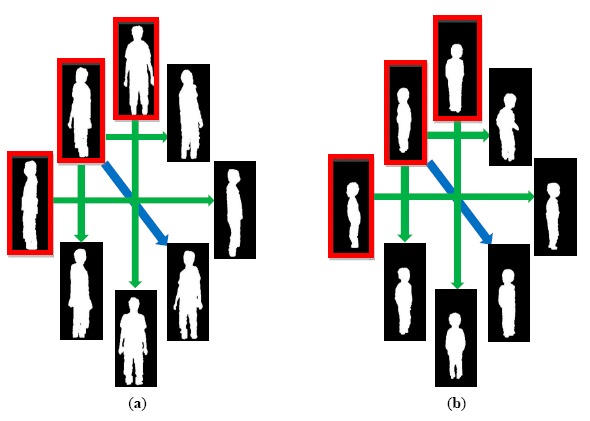
Segmented body silhouettes in various facing directions. (**a**) Adult; (**b**) Child.

In addition to the face images and body colors (FCT), body builds also provide important information for human identification. We modeled a body build as a set of body silhouettes observed from various points of views. The player masks provided by the Kinect's SDK were coupled with skeleton tracking and failed to properly segment human hair as foreground. In our modelling stage, the body silhouettes were segmented by a background subtraction technique solely based on depth images. Because the depth information was invariant to the existence of shadows, the problem of casting shadows was solved inherently. Figure 4 shows the extracted body silhouettes for an adult and a child in multiple views. Three upper-left human silhouettes (in red rectangles) indicate the segmented human masks in three distinct facing directions. Assuming that a human body is symmetric horizontally, human silhouettes in the other five facing directions can be simply obtained by mirroring (shown as green arrow in Figure 4) or cloning (shown as blue arrow in Figure 4). As shown in Figure 1, at least three body silhouettes (front, 45°, and side view) are stored in the appearance database for each registered person.

## 4. Multisensor Human Detection and Tracking

**Figure 5.**
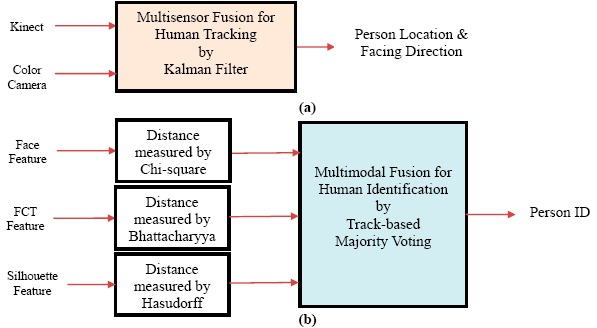
Two data fusion approaches in the proposed system: (**a**) multisensor fusion for human tracking; (**b**) multimodal fusion for human identification.

In the guidance stage, humans can be detected and tracked by a Kinect or a color camera. For a person located at the world coordinate (*x, y, z*), the ground plane coordinate (*x, z*) and the facing direction *θ* of the person were tracked across multiple cameras. Because these cameras can be installed in a wide variety of positions and orientations inside the house, the relationship between the projecting (image) coordinates and world (floor) coordinates should be discovered for each camera. The projective transform between the image plane and the ground plane of the house was unique for each static camera and was computed by four pairs of corresponding points by a camera calibration process in the setup phase. Initially, four points on the floor of the house were specified manually. For each camera, the coordinates of these four points on the ground plane and the four corresponding points on the image plane were utilized to compute a 3 × 3 matrix called homography. With the help of the homography, an image coordinate can be mapped to a ground plane coordinate so that human detected by different cameras can be combined and tracked in a unified ground plane coordinate system. Similarly, the facing direction *θ′* relative to each camera's focal axis can be converted to a global facing direction *θ* before the multisensor fusion. In addition, a multiple camera synchronization was performed using a software-based approach [25]. Finally, the complete or partial measurements from multiple cameras were integrated using a Kalman filter as shown in Figure 5a. It should be noted that our application is not 3D scene reconstruction, thus sophisticated camera calibration (using checkerboard pattern) and synchronization (using hardware genlock) approaches are not required.

### 4.1. Human Detection and Tracking Using a Kinect

Human detection and tracking using a Kinect is straightforward because the Kinect skeleton tracking provides the three-dimensional coordinates of twenty skeletal joints (as described in Section 3). The ground plane coordinate (*x, z*) of a person was extracted from the skeleton joint HIP_CENTER. The facing direction *θ′* was determined in two ways. In a shorter distance where the Kinect face tracking was effective, the facing direction *θ′* was set to the yaw angle of the head pose (as described in Section 3.1); Otherwise, the facing direction *θ′* was set to the angle between the skeletal forward direction and the focal axis of the camera.

### 4.2. Human Detection and Tracking Using a Color Camera

Based on color images acquired by a static camera (either a Kinect or a color camera), moving people were detected by a background subtraction algorithm that integrated the information of color, shading, texture, neighborhood, and temporal consistency [26]. Assuming that a person stands on the floor, the depth *z* between the person and the camera was determined by mapping the person's foot coordinate in the image plane to the ground plane through the aforementioned homography transformation. With the availability of the depth *z* and the focal length *f*, each image plane coordinate (*x′, y′*) can be projected to the world coordinate (*x, y*) using the following equations:

(1) $$\begin{array}{cc} x=z\cdot \frac{x^{\prime }}{f}; & y=z\cdot \frac{y^{\prime }}{f} \end{array}$$

The human body appearance provides additional cues for human tracking and identification. The acquired color images were compared with the registered human templates in the appearance database. A detected person was tracked in the image space using a color-based Mean-Shift approach. The target model was represented by a smaller bounding box (BB) covering the target person and including only the target pixels in the captured color image. The BB was enlarged proportionally to form a larger BB such that the surrounding pixels in the ring area between the larger and smaller BBs were chosen to represent the background. For a feature value *i*, we calculated *p*(*i*) as a normalized histogram of the pixels on the target, and *q*(*i*) as a normalized histogram of the pixels on the background. A log likelihood image [16] was constructed. Subsequently, an iterative Mean-Shift algorithm was performed in the log likelihood image until the BB converged to the location of the target person in the current frame as shown in the upper part of Figure 3. At the same time, the target BB was used as a template to shift its corresponding BB in the FCT as shown in the lower part of Figure 3. In other words, a person was tracked simultaneously on two image domains: horizontally/vertically on the captured image to update image coordinate (*x′, y′*), and horizontally on the FCT image to update facing direction *θ′*. By repeating this process iteratively, the target, background, and FCT models evolved over time all together.

In a typical adaptive tracking procedure with a dynamic update of the target model, the model drift problem appears over time as misclassified background pixels gradually join the foreground model, eventually leading to a tracking failure. To avoid this problem, the histogram of the target model was computed by considering the pixels in the target BB in the current captured image as well as the pixels in the corresponding BB in the FCT. As a result, the target model was adaptive to keep up with the newest conditions. Simultaneously, the target model was constrained by the a priori information in the FCT to prevent accumulation of model drift errors.

### 4.3. Multisensor Human Tracking Based on a Kalman Filter

A person was tracked using a three dimensional vector containing the world coordinate (*x, z*) and the facing direction *θ* across multiple sensors as shown in Figure 5a. From time to time, a sensor can fail to track in some dimensions and produce only partial measurements. It is also possible that multiple sensors observe the same person simultaneously and provide duplicate measurements. To address these problems, a multisensor fusion was performed to accommodate partial and duplicate measured data using a Kalman filter [27]. The four dimensional state vector *X*(*t*) and the three dimensional measurement vector *Z*(*t*) at time step *t* are shown as follows:

(2) $$\begin{array}{cc} X(t)=\left[\begin{array}{c} x(t)\\ z(t)\\ \theta (t)\\ v(t) \end{array}\right], & \text{Z}(t) \end{array}=\left[\begin{array}{c} x(t)\\ z(t)\\ \theta (t) \end{array}\right]$$

where (*x*(*t*), *z*(*t*)) is the ground plan coordinate, *θ*(*t*) is the facing direction, and *v*(*t*) is the velocity on the ground plan of the tracked person at time step *t*. The relationship between the state vector *X*(*t*) and the measurement vector *Z*(*t*) can be formulized as:

(3) $$\{\begin{array}{c} X(t+1)=A(t)\cdot X(t)+w(t)\\ Z(t)=H(t)\cdot X(t)+u(t) \end{array}$$

where *A*(*t*) is the state transition matrix (or called prediction matrix) and *H*(*t*) is the observation matrix (or called measurement matrix). The variables *w*(*t*) and *u*(*t*) are zero-mean white Gaussian noise with covariance matrices *Q*(*t*) and *R*(*t*), respectively. In the proposed human tracking, the Kalman filter was used to produce the optimal state estimate given a sequence of measurements. At each time step, a Kalman filter was applied by an iterative process with two steps. The first step was the time update (or called predictor) that projected forward the current state estimate to obtain an a priori estimate for the next time step. A linear model with a constant velocity was used for the prediction of the human movement, *i.e.*, the state transition matrix was equal to:

(4) $$A(t)=\left[\begin{array}{cccc} 1 & 0 & 0 & \cos(\theta (t))\\ 0 & 1 & 0 & \sin(\theta (t))\\ 0 & 0 & 1 & 0\\ 0 & 0 & 0 & 1 \end{array}\right]$$

The second step was the measurement update (or called corrector) that incorporated a new measurement into the *a priori* estimate to obtain an improved *a posteriori* estimate. The prediction and measurement values were combined according to the prediction and measurement variance. For a complete measurement with three dimensions, the observation matrix was equal to:

(5) $$H(t)=\left[\begin{array}{cccc} 1 & 0 & 0 & 0\\ 0 & 1 & 0 & 0\\ 0 & 0 & 1 & 0 \end{array}\right]$$

In case only partial measurement was obtained (either facing direction or ground plan coordinate was unavailable), the negative impact of the missing data can be cancelled by setting the observation matrix to:

(6) $$\text{either}\;H(t)=\left[\begin{array}{cccc} 1 & 0 & 0 & 0\\ 0 & 1 & 0 & 0\\ 0 & 0 & 0 & 0 \end{array}\right]\;\text{or}\;H(t)=\left[\begin{array}{cccc} 0 & 0 & 0 & 0\\ 0 & 0 & 0 & 0\\ 0 & 0 & 1 & 0 \end{array}\right]$$

The additive nature of the update stage makes the Kalman filter very attractive for multisensor fusion with duplicate measurements. Supposing that there were a set of *N* sensors, *Z_i_*(*t*) was the measurement produced by the *i*-th sensor, and *K_i_*(*t*) was the Kalman gain for the data fusion associated to the *i*-th sensor at time step *t*, the state estimate *X*(*t*) was updated according to the measurements *Z_i_*(*t*) using the following equation [28]:

(7) $$X(t)=X(t)+\overset{N}{\underset{i=1}{\text{∑}}}K_{i}(t)\cdot [Z_{i}(t)-H_{i}(t)\cdot X(t-1)]$$

The covariance matrix *R*(*t*) reflects the uncertainty of the measurements and depends on the characteristics of each sensor. In our implementation, the covariance matrix *R*(*t*) of each sensor was determined by finding the variance in the measurement data that were collected. The Kalman filter was further extended to support multiple people tracking. Supposing that there were *m* maintained tracks and *n* detected persons, an *m* × *n* cost matrix was constructed by computing the cost of assigning every detected person to each track based on the distance between the position of a detected person and the predicted location of an existing track. The cost of assigning a detection to a track was defined as the negative log-likelihood of the detection corresponding to the track. The association problem was solved by generating a detection-to-track assignment which minimized the total costs. In a given frame, some detections might be assigned to existing tracks, while other detections and tracks may remain unassigned. If a detected person was assigned to an existing track, the information of the person was utilized to update the parameters of the assigned track by the Kalman filter; otherwise, a new track was created for the unassigned person. Tracks that have been continuously updated for a fixed number of frames were classified as steady. Oppositely, tracks that have not been updated for a fixed number of frames were discarded.

## 5. Multimodal Human Identification

Human can be identified by comparing the color images (captured by either a Kinects or a color camera) with the registered templates in the appearance database using an individual modality of the face, body color, or silhouette. As shown in Figure 5b, a decision level fusion was applied for the human identification to adopt the most suitable feature set, distance measure, and identification method for each single modality. Afterwards, the identification outputs were integrated by a track-based majority voting to make a final identification decision.

### 5.1. Human Identification Using Faces

Face recognition can be performed if the detected person faces the camera within a close range. Eigenfaces [19] and Fisherfaces [20] are holistic approaches that work in a high-dimensional image space and require several facial images for each person to achieve good recognition rates. Alternatively, we adopted local binary patterns (LBP) which summarized the local structure in a facial image by comparing each pixel with its neighborhood. By definition the LBP operator is robust against illumination changes. After the location and size of the face of the captured person were determined, the cropped face was aligned to upright pose and normalized to a standard size. Subsequently, the LBP image was divided into local regions and a histogram was extracted from each region. The spatially enhanced feature vector, called LBPH [29], was then obtained by concatenating the histograms of LBPs. The 32 most relevant features selected through PCA were used to recognize a face by comparing a captured face with each registered face template in the appearance database. After comparing various distance (or dissimilarity) measures, we found that the best distance measure for the comparison of LBPH between histograms *S* and *M* was a Chi-square statistic: *x*^2^(*S,M*) = ∑*_i_*[(*S_i_*−*M_i_*)^2^/(*S_i_*+*M_i_*)].

### 5.2. Human Identification Using FCTs

Because a clear facial image is not always available, the human body appearance provides additional cues for the human identification. A detected person was tracked using a color-based Mean-Shift approach described in Section 4.2, and represented as a bounding box (BB). To match color information in the detected BB and a BB of a FCT in the appearance database, a histogram was divided to 32 bins and each bin covered a horizontal stripe of the human body. After comparing various color spaces and distance measures, we found that the YC_b_C_r_ color space and the Bhattacharyya distance performed the best in the process of the Mean-Shift tracking and color histogram matching. The Bhattacharyya distance between two histograms *S* and *M* was defined as $\beta (S,M)={\text{∑}}_{i}\sqrt{S_{i}\cdot M_{i}}$. Similar to the human tracking, the target model was compared with each FCT in the appearance database using the same distance measure to find the best match for human identification as shown in the lower part of Figure 3.

### 5.3. Human Identification Using Silhouettes

In addition to face images and body colors (FCT), body shape information also provides distinctive clues for the human identification. In the guidance stage, human silhouettes were extracted by the background subtraction. To compare a detected silhouette mask with each silhouette template in the appearance database, two contours were scaled, aligned, and matched. First, the size of a 2D contour was normalized to reflect its real size in 3D. Each segmented human silhouette was scaled by a factor that was inversely proportional to the person's depth *z* that was determined in the aforementioned homography computation. Second, two contours were translated so their barycenters overlapped, and rotated so their major axes aligned. Third, a polar coordinate space was equally divided into 32 sectors and each human silhouette was sampled as a 32 dimensional vector accordingly. Finally, the Hausdorff distance [30] between two silhouettes was computed. Given one set of points *A* containing pixels along the boundary of a detected silhouette and another set of points *B* containing pixels along the boundary of a template silhouette, the Hausdorff distance provided a mean to determine the resemblance of these two set of points and was defined as the greatest of all the distances from a point in *A* to the closest point in *B*. To compare a portion of shapes for partial shape matching, the *K^th^* ranked distance was selected instead of the maximal distance and the constant *K* controlled how many points of the model needed to be near points of the target, *i.e., H*(*A,B*) = max(*h*(*A,B*),*h*(*B,A*)), where $h(A,B)=\underset{a\in A}{K^{th}}\min_{b\in B}\left\|a-b\right\|$. The Hausdorff distance can be computed efficiently and can handle translation, scaling, and partial shape matching effectively.

### 5.4. Multimodal Human Identification Based on a Majority Voting

At a time step, the human identification was performed individually using face, FCT, and silhouette modalities. Face recognition was possible if facial images can be captured clearly within a close range. Adding appearance information of body colors (FCT) and the body shapes (silhouette) further promoted the recognition accuracy. In each aforementioned track (described in Section 4), the identification results of these three modalities (face, FCT, and silhouette) were accumulated over time using a majority voting for human identification as shown in Figure 5b. To measure the quality of the identification result of each modality in each frame, a confidence value was defined as *C_d_* = (*d_2_* − *d_1_*)*/d_2_* where *d_1_* was the distance of the best match and *d_2_* was the distance of the second best match. The confidence value *C_d_* ranged from zero to one. The greater the confidence value *C_d_* was, the higher the probability was of the fact that the best match with the shortest distance was the actual match. The identified person ID of each modality in each frame can vote with a weight that was equal to its confidence *C_d_*. The person ID receiving the maximal number of votes was outputted as the final identification result of the multimodal fusion. Compared to a frame-based identification, the track-based identification with a long-term majority voting was much more reliable.

### 5.5. Multimodular Cooperation

**Figure 6.**
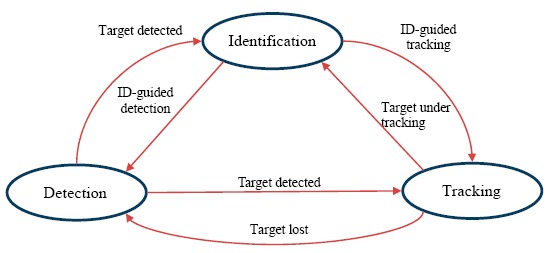
Cooperation among detection, tracking, and identification modules.

As shown in Figure 6, the detection, tracking, and identification modules can cooperate seamlessly and benefit from each other to achieve a robust system. The detector treated every frame independently and performed a full scan of the image to localize potential foreground people. To reduce false alarms made by the detector, the tracker estimated the detected person's motion between consecutive frames under the assumption that the frame-to-frame motion was limited. Nevertheless, the tracker was likely to fail if the tracked person moved out of the camera's view.

In this case, the detector discovered any newly appearing person, then re-initialized the tracker, and thus minimized the tracking failures. Moreover, the identification module can improve the tracking performance by constraining the target model by the registered FCT of the identified person to avoid model drift errors in the adaptive update process. Also, the identification module can help the detector to reduce miss-detections by providing color distributions of the recent identified person for better discrimination between the foreground and background in the process of the background subtraction.

## 6. Evaluations

**Figure 7.**
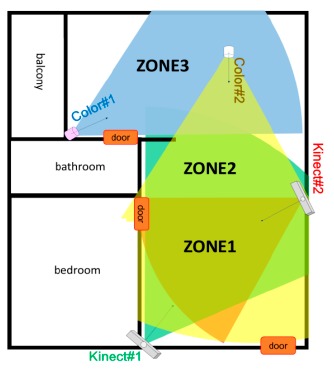
Typical layout of an elderly apartment. Two Kinects and two color cameras were installed to cover most open areas in the apartment.

Figure 7 shows a typical layout of an elderly apartment. Two Kinect were installed on opposite sides with overlapping field of view (FOV) in the living room for modelling the multi-view full-body appearance, and two color cameras were installed to cover the views of the other parts of the apartment for human detection, tracking, and identification.

**Figure 8.**
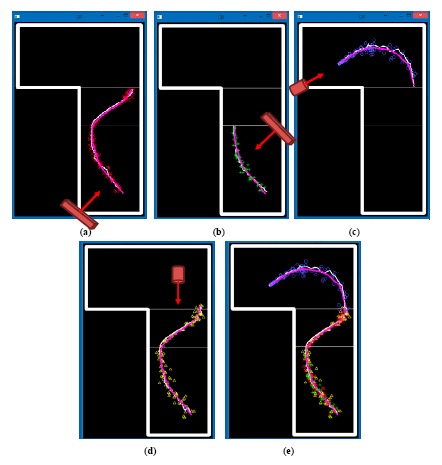
Measurements of distinct sensors: (**a**) red cross mark for Kinect#1; (**b**) green plus mark for Kinect#2; (**c**) blue circle mark for color#1; (**d**) yellow triangle mark for color#2; (**e**) multisensor fusion; Thin white curve indicates the Kalman estimated trajectory, and thick purple curve represents the actual trajectory (ground truth).

The first experiment evaluated the effectiveness of the multisensor human tracking using a Kalman filter. Five people walked around in the open space in the apartment in turn and each individual track on the ground plan was marked manually as the ground truth. A total of 100 tracks (150 frames in each track) were recorded and tracked across two Kinects and two color cameras. For evaluation purpose, a measurement error $E_{f}^{M}$ and a Kalman estimate error $E_{f}^{K}$ for each tracking feature dimension *f* (*f* = *x, z*, or *θ*) were defined using mean square error (MSE):

(8) $$\begin{array}{l} E_{f}^{M}=\sqrt{\frac{1}{m\times n}\cdot \overset{m}{\underset{T=1}{\text{∑}}}\overset{n}{\underset{t=1}{\text{∑}}}{\left[G_{f}(T,t)-M_{f}(T,t)\right]}^{2}}\\ E_{f}^{K}=\sqrt{\frac{1}{m\times n}\cdot \overset{m}{\underset{T=1}{\text{∑}}}\overset{n}{\underset{t=1}{\text{∑}}}{\left[G_{f}(T,t)-K_{f}(T,t)\right]}^{2}} \end{array}$$

where *m* is the number of tracks, *n* is the number of frames in each track; *G_f_* (*T, t*) represents the ground truth, *M_f_* (*T, t*) indicates the measurement, and *K_f_* (*T, t*) stands for the Kalman estimate of the feature dimension *f* in the *t*-th frame in the *T*-th track. For a typical track in the apartment, Figure 8 plots the measurements of distinct sensors (red cross marks for Kinect#1, green plus marks for Kinect#2, blue circle marks for color#1, and yellow triangle marks for color#2). The final estimated trajectory after multisensor fusion using a Kalman filter is shown as a thin white curve, and the actual trajectory (ground truth) is shown as a thick purple curve in Figure 8e.

As shown in Figure 7, the open space in the apartment can be divided to three zones. The first zone (ZONE1, lower part in Figure 7) was monitored by three cameras (Kinect#1, Kinect#2, and Color#2). The second zone (ZONE2, middle part in Figure 7) was monitored by two cameras (Kinect#1 and Color#2). The third zone (ZONE3, upper part in Figure 7) was monitored by a single camera (Color#1). Table 2 compares the tracking errors of measurements (before tracking) and Kalman estimates (after tracking) using distinct sensor types. Generally, the measurement errors of Kinects were lower than which of color cameras, and the measurement errors of the feature dimension *z* were higher than which of feature dimension *x*. The Kalman filter effectively reduced the tracking error for both Kinect and color camera. Table 3 compares the tracking errors of measurements (before tracking) and Kalman estimates (after tracking) combining different number of sensors. It can be noted that the negative effects caused by wrong or missed measurements were suppressed by the multisensor fusion using the Kalman filter. Compared to a Kalman tracker using a single sensor (ZONE3), a Kalman fusion of two sensors (ZONE2) made an improvement of 21.4%, 37.4%, and 24.6% of the feature dimension *x, z*, and *θ*, respectively; a Kalman fusion of three sensors (ZONE1) achieved an improvement of 38.8%, 51.0%, and 46.0% of the feature dimension *x, z*, and *θ*, respectively.

The second experiment evaluated the reliability of the multimodal human identification using the proposed majority voting. Five family members in an apartment were involved: an elderly male (ID#1), a young adult male (ID#2), a young adult female (ID#3), a teenage female (ID#4), and a toddler male (ID#5). Tables 4 – 6 show the vote matrices of human identification of the proposed system solely based on faces, FCTs, and silhouettes, respectively. Each number in a vote matrix indicated the number of votes of a specific person ID in an individual track, each column represented an identified person ID by the proposed system, and each row corresponded to an actual person ID in the track. All correct votes were located in the diagonal of a vote matrix. Similar to the aforementioned confidence value of the identification result of each modality in each frame, the confidence value of the identification result of a track was defined as *C_v_* = (*v_1_* – *v_2_*)*/v_1_* , where *v_1_* was the highest number of votes and *v_2_* was the second highest number of votes. The confidence value *C_v_* ranges from zero to one. The greater the confidence value *C_v_* is, the higher the probability is of the fact that the identification result with the highest votes is the actual ID. The number with the highest votes was emphasized in bold font, and mismatches (the winner ID not equal to the actual ID) were annotated with an exclamation mark. Identification solely based on faces tended to fail if clear face images were unavailable; Identification solely based on FCTs could be confused by clothes with similar colors and patterns; Identification solely based on silhouettes was ineffective to differentiate people with similar body builds. Table 7 shows the vote matrix of the proposed human identification considering faces, FCTs, and silhouettes all together. With the proposed multimodal fusion, reliable identification results were produced with high confidence values.

**Figure 9.**
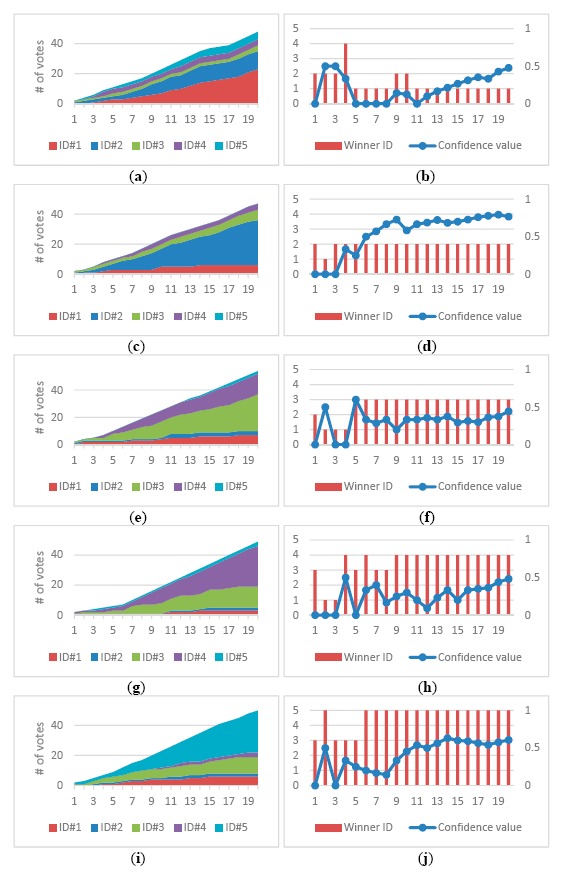
Track-based voting for the human identification in five tracks with distinct IDs. (**a**) the voting result over time in the first track; (**b**) the winner ID with the highest votes and its confidence value over time in the first track; (**c**–**j**) for the second∼fifth track.

Figure 9a shows the voting results of a track over time. Three modalities of faces, FCTs, and silhouettes voted independently to identify a detected person in the appearance database registered with five people. At the beginning of the track, several ID competed with each other and the computed confidence was low. As the track continues, more votes come in and ID#1 gradually got ahead over time. Finally, ID#1 dominated the vote at the end of the track and the detected person was identified as ID#1 with high confidence value. Figure 9b shows the changes of the winner ID with the highest votes and its confidence value of a track over time. Figure 9c–j shows the voting results of the other four tracks. With the accumulation of votes in each track, the proposed human identification gradually obtained more reliable results with higher confidence values over time. As shown in the third and fourth tracks, individual identification modality tended to confuse between ID#3 and ID#4 because they have similar faces and body builds. Nevertheless, the FCT modality can differentiated them well, and gradually accumulated enough votes to make a correct identification decision at the end.

**Figure 10.**
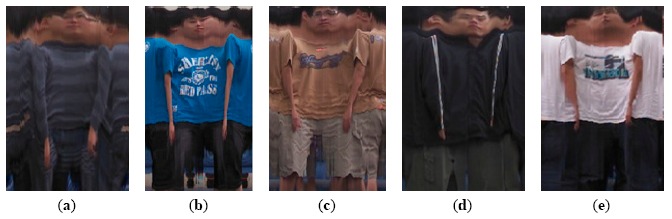
Constructed FCTs of five adult males with similar body builds.

The third experiment compared the performance of the proposed multimodal identification, Gandhi's full-body appearance identification [21], and Ahonen's face recognition [29] in an apartment with two Kinects and two cameras. Gandhi's method was implemented to identify full-body appearance samples which were obtained from all visible cameras and integrated using registration and temporal averaging described in their paper. Ahonen's method was implemented to recognize frontal face samples whenever they were detected within a close range of any visible camera. To make a fair comparison, identification results of each method were accumulated over time using the proposed majority voting (described in Section 5.4) to make a track-based final decision. Table 8 shows the recognition rates using different methods in various scenarios. The first scenario was under normal conditions in that ten participants walked around in the apartment one at a time. Their FCTs were constructed and stored in the appearance database using the image mosaicking process described in Section 3.2. The second, third, fourth, and fifth scenarios were under more challenging conditions. The second scenario consisted of five adult males with similar body builds (constructed FCTs as shown in Figure 10). Even if the silhouette modality failed to make a distinction, the other modalities (FCT and face) still worked and helped to make a correct final decision in the process of the majority voting. The third scenario consisted of two females with similar faces. There was some ambiguity using Ahonen's face recognition due to the resemblance of face features. The proposed multimodal method outperformed theirs by utilizing more modalities (FCT and silhouette). The fourth scenario consisted of three persons wearing similar colored dresses. Gandhi's method was confused by the small distance measure between full-body appearance samples with similar color layouts. The proposed multimodal recognition correctly disambiguated them by the other modalities (face and silhouette). The fifth scenario evaluated the robustness of the multiple people tracking described in Section 4.3, and compared the recognition rate using different methods. Even if two or three persons appeared in the scene simultaneously, the result indicated that the proposed multimodal identification was robust under multiple people conditions. The problem of visual occlusions between two persons was alleviated in the proposed multisensor environment with various perspectives and the multimodal fusion.

**Figure 11.**
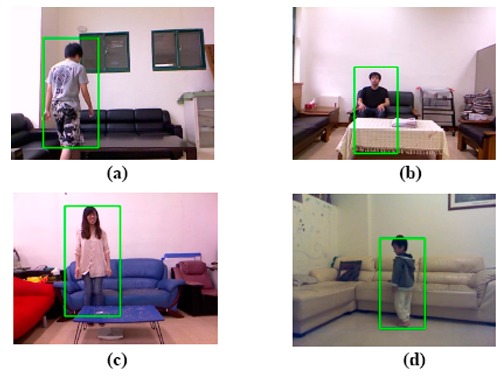
Example images captured in four living rooms in different apartments.

The fourth experiment involved ten participants in four different apartments (as shown in Figure 11). The goal of the experiment was to collect feedback from real users. The participants were interviewed regarding their experiences using the proposed human detection, tracking, and identification system at their homes. Their comments indicated the strengths and weaknesses of the proposed system and provided directions of further improvements. Even if cameras were not installed inside the bedroom or bathroom, several participants expressed their concerns regarding the privacy. Under normal conditions, the captured images can be immediately destroyed once a track is finished. In case an emergency is detected, the captured images can be stored for record purpose only. The database and the captured images will not be shared with anyone without authorization. Also, family members can turn off any cameras at any time. Another concern is the correctness of the automatic modelling stage. Because a set of multiple FCTs can be stored for each person to remember their favorite leisure wears at home, a FCT scan is performed and recorded in the appearance database semi-automatically if a new suit of clothes is detected. Human intervention is only required in scheduled maintenance to correct any possible misplacement made by the automatic modelling process. Examples of comments regarding these problems are as follows:

“Can I turn off the cameras by myself?”

“I don't want to broadcast live videos of my personal space on Internet”

“Do I need to register again after I change clothes at home?”

Despite these problems, participants expect the system to provide useful information on their daily routines at home. Even if no camera was installed inside the bathroom, each door of the bathroom or bedroom was in the FOV of at least one camera. The proposed human detection, tracking, and identification system can count the number of times a specific person enter the bathroom per day by monitoring the image area around the door (marked as red rectangle in Figure 7). Similarly, the system can also record the time a specific person enter or leave the bedroom for a rough measurement of sleep amount. The comments from participants indicated that they anticipate the system to provide more intelligent services in the future:

“Can the system count how many calories I burned by walks at home per day.”

“Will the system warn me promptly if my elders or kids fall at home?”

“My doctor wants to know how many times I go to the bathrooms per day.”

“It is nice to know the system will inform me if an intruder is detected.”

## 7. Discussion

Although a Kinect can detect, track, and identify people reliably, its working range is limited. The FOV of a Kinect is 43° vertically and 57° horizontally. The Kinect's specifications show that the optimal distance between a Kinect and its target is about 0.8 ∼ 4.0 m. Our experiences during the experiments indicated that the Kinect skeleton tracker was reliable in a three-dimensional frustum with a size of about 4.0 m (*x*-axis) × 3.0 m (*y*-axis) × 3.0 m (*z*-axis). The working distance of the Kinect's face tracker is about 1.0∼3.0 m. The face tracker is robust under three-dimensional head rotations with a yaw range of ±60° and a tilt range of ±45°. Relatively, the working distance of the color camera is not limited. The FOV of the color camera is 40° vertically and 51° horizontally.

Partial occlusions occur when some body parts are occluded by closer objects (such as furniture) and usually happen on the human lower body. The up-to-date Kinect SDK (Version 1.5 or newer) provides two tracking modes that can be switched dynamically at run time. The normal tracking mode is optimized to recognized and track people who are standing and fully visible. On the other hand, the seated tracking mode focuses on the tracking of the human upper body in case the lower body was visually occluded (as shown in Figure 11c), or was seated on a couch (as shown in Figure 11b). We designed an algorithm to switch between the normal and seated modes automatically by analyzing the matching scores of the color histogram of the detected person and which of the FCT in the database. As described in Section 5.2, each histogram bin covering a horizontal stripe of the human body can produce an individual matching score. If the matching scores in the histograms of the lower body reduced suddenly, it indicated that the lower body was occluded and the tracking system switched to the seated mode to concentrate on tracking the upper body. Conversely, if the matching score of the lower body returned to the normal range (similar to that of the upper body), it indicated a leaving of occlusion state and the tracking system returned to the normal mode to track the complete body. Dynamic switching between these two modes prevented drift-over-time errors of the tracking in scenarios with seated postures or lower body occlusions.

The proposed human detection, tracking, and identification system was designed for general purpose at digital home and provided the foundation of further analysis for intelligent healthcare services. The symmetry of human posture, smoothness of moving trajectory, and variation of walking pace can be analyzed to provide important visual cues to assess fall risks. Also, the segmented human silhouettes can be classified for accidental fall detection. Besides, the captured facial images can also be analyzed to recognize abnormal facial expression such as pain or twitch. In the future, we propose to explore the possibilities to provide early warning or call for help automatically in case of anomalies or emergencies. Furthermore, the proposed system has potentials to assist family members with health control in daily life by measuring physical activity amount or recording sleep time.

## 8. Conclusions

As information and communication technologies (ICT) continue to advance, smart digital homes promise to provide us with safer, more comfortable and economical residences. To provide intelligent and personalized healthcare services in daily life at home, we have proposed an appearance-based surveillance system to detect and track where the person locates, and to identify who the person is. A set of Kinects were installed at home entrance to model and record multi-view faces, body colors (FCTs), and shapes (silhouettes) of family members in an appearance database. A different set of color cameras were installed to cover the views of the other parts of the apartment. An adapted Mean-Shift algorithm was performed for *human tracking* on both captured image and FCT in the database simultaneously. The target model of tracking was updated iteratively over time to accommodate multi-view variations but still constrained by the a priori information in the FCT to avoid model drift problem. A Kalman fusion of multiple sensors (Kinects and color cameras) was applied for human tracking on the ground plane. The multi-view *human identification* was solved by a multimodal fusion using a track-based majority voting that matched the detected person with the registered face, FCT, and silhouette templates in the appearance database. With independent operations, the tracker, detector, and identification modules can fail occasionally. A multimodular integration was proposed so the detection, tracking, and identification modules can benefit from each other and cooperate seamlessly. The proposed human detection, tracking, and identification system can be extended to provide more intelligent services in home security and home healthcare.

## Figures and Tables

**Table 1.** Comparison of various sensors for human detection, tracking, and identification at home.

	**RFID-Based Technology**	**Vision-Based Technology**			
		**Fingerprint**	**Iris**	**Face**	**Body Appearance**
**Hardware Requirement**	RFID reader, tag	Fingerprint scanner	Iris scanner	Close-up camera	Camera
**User Intrusion**	Wear RFID tag	Provide finger	Show eye	Turn to face camera	Non-intrusive
**Detection under Various Poses**	Yes	No	No	Limited by angle	Yes
**Identification Accuracy**	Absolute	High	Highest	Medium-high	Medium-high
**Dynamic Illumination**	Unaffected	Unaffected	Infrared better	Shadow, lighting	Shadow, lighting
**Visual Occlusion**	Unaffected	No	No	Limited	Partial
**Multi-View Tracking**	Not accurate	No	No	Limited by angle	Yes

**Table 2.** MSE errors of human tracking using different sensor types in each feature dimension: *x, z*, and *θ*; the unit is cm, cm, and degree, respectively.

**Sensor Type**	**Measurements**			**Estimates**		
	
	**$E_{x}^{M}$**	**$E_{z}^{M}$**	**$E_{\theta }^{M}$**	**$E_{x}^{K}$**	**$E_{z}^{K}$**	**$E_{\theta }^{K}$**
Kinect	5.70	8.06	10.45	4.43	3.39	6.67
Color Camera	7.80	9.85	15.92	6.28	5.94	11.33

**Table 3.** MSE errors of multisensor human tracking using different number of sensors in each feature dimension: *x, z*, and *θ*.

**Number of Sensors**	**Measurements**			**Estimates**		
	
	**$E_{x}^{M}$**	**$E_{z}^{M}$**	**$E_{\theta }^{M}$**	**$E_{x}^{K}$**	**$E_{z}^{K}$**	**$E_{\theta }^{K}$**
1 (ZONE3)	8.18	10.33	16.83	6.13	6.23	11.50
2 (ZONE2)	5.12	4.90	14.80	4.82	3.90	8.67
3 (ZONE1)	6.98	9.71	13.35	3.75	3.05	6.21

**Table 4.** Vote matrix of the proposed human identification solely based on Face.

**Recog. ID**	**#1.**	**#2**	**#3**	**#4.**	**#5**	**Winner ID**	**Confidence Value**

**Real ID**							
#1	5	4	0	2	0	#1	20%
#2	2	6	0	2	0	#2	67%
#3	0	1	10	4	0	#3	60%
#4	1	0	7	6	0	#3(!)	14%
#5	2	0	0	3	7	#5	57%

**Table 5.** Vote matrix of the proposed human identification solely based on FCT.

**Recog. ID**	**#1**	**#2**	**#3**	**#4**	**#5**	**Winner ID**	**Confidence Value**

**Real ID**							
#1	11	0	3	0	5	#1	55%
#2	0	17	1	0	0	#2	94%
#3	3	0	11	4	2	#3	64%
#4	2	0	4	10	3	#4	60%
#5	3	2	9	0	5	#3(!)	44%

**Table 6.** Vote matrix of the proposed human identification solely based on Silhouette.

**Recog. ID**	**#1**	**#2**	**#3**	**#4**	**#5**	**Winner ID**	**Confidence Value**

**Real ID**							
#1	7	8	1	2	0	#2(!)	13%
#2	4	7	6	2	0	#2	14%
#3	4	2	6	7	0	#4(!)	14%
#4	0	2	3	11	0	#4	73%
#5	1	0	2	0	16	#5	88%

**Table 7.** Vote matrix of the proposed human identification based on a multimodal fusion.

**Recog. ID**	**#1**	**#2**	**#3**	**#4**	**#5**	**Winner ID**	**Confidence Value**

**Real ID**							
#1	23	12	4	4	5	#1	48%
#2	6	30	7	4	0	#2	77%
#3	7	3	27	15	2	#3	44%
#4	3	2	14	27	3	#4	48%
#5	6	2	11	3	28	#5	61%

**Table 8.** Comparison of the recognition rate using different methods in various scenarios.

**Scenarios**	**#1**	**#2**	**#3**	**#4**	**#5**
Participants	10 persons, one at a time	5 adult males with similar body builds	2 females with similar faces	3 persons wearing similar colored dresses	2 or 3 persons in the scene simultaneously
# of tracks	100	50	20	30	10
Recog. Rate of the Proposed multimodal method	96%	92%	95%	87%	90%
Recog. Rate of Gandhi's full-body recognition [21]	89%	88%	95%	63%	80%
Recog. Rate of Ahonen's face recognition [29]	74%	76%	60%	70%	70%
